# A lattice model to manage the vector and the infection of the *Xylella fastidiosa* on olive trees

**DOI:** 10.1038/s41598-019-44997-4

**Published:** 2019-06-19

**Authors:** Annalisa Fierro, Antonella Liccardo, Francesco Porcelli

**Affiliations:** 1 Consiglio Nazionale delle Ricerche (CNR) - Institute Superconductors, oxides and other innovative materials and devices (SPIN), Napoli, Italy; 2 Physics Department, Università degli Studi di Napoli “Federico II”, Napoli, Italy; 3 Istituto Nazionale Fisica Nucleare (INFN) - Sezione di Napoli, Napoli, Italy; 4 Dipartimento di Scienze del Suolo, della Pianta e degli Alimenti, University of Bari Aldo Moro, Bari, Italy

**Keywords:** Statistics, Ecological epidemiology

## Abstract

Since October 2013 a new devastating plant disease, known as Olive Quick Decline Syndrome, has been killing most of the olive trees distributed in Apulia, South Italy. *Xylella fastidiosa pauca* ST53 is the plant pathogenic bacterium responsible for the disease, and the adult Meadow Spittlebug, *Philaenus spumarius* (L.) (Hemiptera Aphrophoridae), is its main vector. This study proposes a lattice model for the pathogen invasion of olive orchard aimed at identifying an appropriate strategy for arresting the infection, built on the management of the vector throughout its entire life cycle. In our model the olive orchard is depicted as a simple square lattice with olive trees and herbaceous vegetation distributed on the lattice sites in order to mimic the typical structure of an olive orchard; adult vectors are represented by particles moving on the lattice according to rules dictated by the interplay between vector and vegetation life cycles or phenology; the transmission process of the bacterium is regulated by a stochastic Susceptible, Infected and Removed model. On this baseline model, we build-up a proper Integrated Pest Management strategy based on tailoring, timing, and tuning of available control actions. We demonstrate that it is possible to reverse the hitherto unstoppable *Xylella fastidiosa pauca* ST53 invasion, by a rational vector and transmission control strategy.

## Introduction

Olive Quick Decline Syndrome (OQDS) is a severe plant disease, spreading northward in Apulia, South Italy since 2013, and causing the death of most of the olive trees distributed in more than 23,000 ha of olive orchards. Due to the relevance of olive production and olive industry in the Mediterranean region, this outbreak caused major environmental and economic damage, endangering the income of thousands of families in that rural area of South Italy. OQDS is nowadays considered a phytosanitary priority for all EU countries.

Sudden symptoms of OQDS range from leaf scorching to desiccation of leaves, fruits, and twigs. Deciduous plant organs do not drop-down, but stay on the twigs because drying is so fast to impede physiological abscission. The focal sectorial symptoms appear on top twigs, spreading down to the whole canopy, in response to severe evapo-transpirative stresses. The symptoms appear after 18–24 months from infection, driving the olive trees to death in 2–5 years. The pruning, even when followed by new shoots arising, does not allow the tree to survive.

The OQDS firstly appeared in Italy between 2008–2010 in a restricted region near Gallipoli (LE), and the first suspicions fell on few already known pest and pathogens as the leopard moth (*Zeuzera pyrina*), and the anthracnose (*Colletotrichum spp*.) and other fungi of different families (*Phaeoacremonium*, *Phaemoniella*, *Pleumostomophora*, *Neofusicoccum*^1,2^). The need to identify the plant pathogen responsible for the OQDS became urgent during 2013. Studies conducted by the University of Bari and CNR drew the attention on the *Xylella fastidiosa*, detecting its presence on most of the symptomatic trees^3^. Nowadays, it is almost accepted that this bacterium is responsible for the OQDS in Southern Italy^4^. The bacterium invades the tree xylem, impeding the raw sap flow from the roots to the leaves and slowly leading the tree to death.

*Xylella fastidiosa* was firstly identified in the Americas, where it was associated with a disease of grapevine in USA^5^ and of citrus trees in Brazil. It appears in 4 different subspecies (*fastidiosa*, *multiplex*, *sandyi* and *pauca*), the one associated to the symptomatic olive trees in Italy being the subspecies *pauca*^6^ - sequence type 53 (ST53) - as demonstrated by genomic studies^7^. It is believed that this strain could have been introduced in Europe by the trade of ornamental asymptomatic Coffee plants from Costa Rica^8^, where the same genotype was previously identified. The actual distribution of *Xylella fastidiosa* depends on: (1) intercontinental trade of infected plant material, (2) transfer by vectors, (3) differentiation of the bacterium in many diverse subspecies and strains with different pathogenic attitudes, which make them relevant for different host plants.

*Xylella fastidiosa* is transmitted by xylem sap-feeder vector, which acquires the bacterium while feeding from infected trees. Then the bacterium multiplies into the vector foregut lumen, which is external to the vector body, and for this reason it is immediately re-transmittable with no latency, by the vector’s stylets in tissues of other healthy or already infected trees during the subsequent feedings. It has been ascertained^9^ that the principal vector of the *Xylella fastidiosa*, in the OQDS outbreak in Apulia, is the meadow splittlebug, *Philaenus spumarius*, which is indeed the most abundant xylem-fluid feeding insect in olive orchards^10,11^. There are other two species capable to transmit the ST53 strain, the *Philaenus italosignus* and the *Neophilaenus campestris*, which are less effective vectors, even if their real ability to transmit is not quantified today^12^. For the purpose of this study, we do not distinguish among different species and assume all of them to have the same acquisition and transmission attitude of the *Philaenus spumarius*.

**Figure 1:**
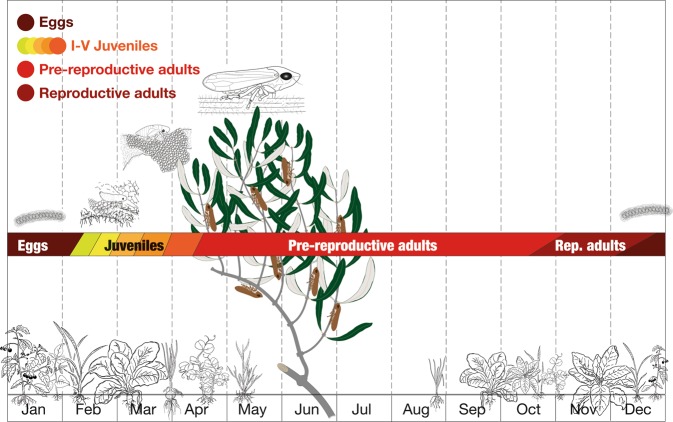
Vector life-cycle in relation to host plants phenology in olive orchard.

The life cycle of *Philenius spumarius* is one year long and the vector phenology is locally influenced by the annual weather fluctuation and, globally, by climatic factors. Figure 1 shows the *Philenius spumarius* life cycle under typical climate conditions of Southern Italy. Eggs are laid in early winter and hatching occurs during February. There are five juveniles instars (three naiades, plus two nymphs) anticipating the imago (adult) eclosion. We stress that naiades are different from nymphs because the former miss the wing buds. Naiades start to inhabit herbs and weeds in olive orchards, secreting mucus and frothing the typical spittle. Juvenile vectors are almost immotile on spontaneous vegetation until the half of April, when the eclosion occurs. After the eclosion, adult spittlebugs promptly move away from drying herbs to infest the nearest growing-up plants, in particular olive trees. The life cycle of the vector is interconnected with the phenology of vegetation. In particular herbaceous vegetation undergoes a seasonal cycle as well, with a drying phase during the spring-summer and a thriving phase in the remaining part of the year. Analogously, after flowering, olive twigs harden, becoming impenetrable to the vectors. The interplay between the vector and vegetation life cycles (see Fig. 1) influences the feeding preferences of the vectors, and is responsible for the seasonal overall displacement of the population from vegetation to trees and vice versa.

There are a few models in literature developed in order to predict the spreading of this infection, to inform control policies and furnish guideline for the surveillance system based on spatially explicit simulation model^13^, or on network approaches^14^ combined with machine learning techniques^15^, or based on Bayesian inference^16^. We believe that any model aimed at identifying the appropriate strategy to manage the infection should consider the life cycle of the vector and provide for diverse appropriate control means and actions at each of its life instars, within an Integrated Pest Management (IPM) strategy.

In this paper we construct a quantitative lattice model for the infection spreading. The adoption of a lattice model to describe the spreading of an infection was already successfully employed in a series of paper^17–20^ in the case of human H1N1 outbreak. In the present study, the olive orchard is constructed as a square lattice, with olive trees and herbaceous vegetation distributed on the lattice sites in a realistic way. Adult vectors are depicted as particles moving on the lattice according to specific rules dictated by the vector life cycle and feeding preferences during the year. The transmission process of the *Xylella fastidiosa* by the vectors is regulated by a stochastic Susceptible, Infected and Removed (SIR) model, with no delay in acquisition and no latency in transmission.

The purpose of this study is to show that an appropriate vector and transmission IPM strategy, based on a few key actions with an appropriate timing, can mitigate or even stop the plant pathogen invasion. One should keep in mind that vectors can acquire and transmit as adult only during a narrow transmission window, whereas juveniles are non-transmitting individuals. This circumstance makes it possible to target the juveniles first, by several population control actions, and then to decimate the adults before they acquire or at the time of their first acquisition. Thus, in order to reduce the overall population, it is firstly necessary to act against eggs and juveniles. Actually there is a set of control actions that can be imposed with purposely-built distribution machines, with an appreciable contribution to the transmission management. This IPM requires the accurate timing of juvenile control action at the passage from last naiad to first nymph to be effective.

Next, further control actions against adults are necessary to prevent and protect the plants from the pathogen transmission. A preventive and protective IPM strategy was already proposed to solve the *Rhyncophorus ferrugineus* (Coleoptera Curculionidae) invasion on *Phoenix canariensis* (Arecaceae). Such a kind of approach is needed for the management of a lethal infestation/disease with delayed damages/symptoms appearance^21,22^. By the term transmission, we indicate the action of an adult vector that has already acquired *Xylella fastidiosa* and that passes the bacterium to a plant by feeding. Three cases are possible: (1) the first transmission (the infection) of *Xylella fastidiosa* to a hitherto uninfected plant, (2) the auto-infection (i.e. an intra-infected plant transmission), (3) the allo-infection (i.e. the inter-infected plant transmission). The first event permits the plant pathogen invasion. Nevertheless, auto- and allo-infections accelerate and aggravate the symptomatology by increasing the bacterial population and, thus the OQDS. Although we have several population management actions to opt and sort, the transmission management can today rely only on chemical control action on olive trees, accurately timed and eventually repeated on short time-interval to prevent the possibility for an adult to feed two times on olive trees. In other words, the chemical action should kill the adult at the time of its first feeding on the tree, despite the olive tree is infected or not, annihilating the otherwise following *Xylella fastidiosa* transmission to the same or other trees. The approach to transmission management is then preventive and protective: it prevents the infection, because a vector dies at the time of the eventual acquisition from an infected plant, and protects all the plants allowing one or few transmissions during the adult life-cycle, purposely shortened. Protection to still uninfected plants is also due to the “lure and kill” role that infected, but not yet symptomatic, treated olive trees play when a vector comes to them for feeding. Whether a vector is infected or not, the feeding on an insecticide-treated tree will provoke the death of the vector and qualify the olive tree as a trap-plant that reduces the number of future transmission, without the risk to augment the actually infected plants. Olive trees can be protected from transmitting vectors by preventive spray or injection distribution of synthetic xylem-moving insecticide, chosen on the basis of experimental mortality data^23–26^.

Summarising, we can describe the egg and juvenile population management as control key points aiming at diminishing the population size by eliminating a percentage of individuals. All the same, the transmission management is a control key point aiming at minimising the pathogen acquisition and the following infections. None of these actions can be disregarded to obtain the IPM functional response over the management of both the vectors and the plant pathogen invasion.

In our model, eggs and juvenile populations, eventually reduced by appropriate control actions^27^, fix the initial conditions of the numerical experiments, in which the adult dynamics and the resulting *Xylella fastidiosa* transmission on an olive orchard are simulated. Wishing to be extremely cautious, we choose to set the parameters of the model at their worst efficacy values, getting the minimum possible vector and transmission control success. Our decision lies on the consciousness that in the real word the pest control actions are often imposed in a long far than optimal way. We show that, even adopting conservative estimates for the efficacy of the control actions, it is always possible to reduce the *Xylella* invasion, and its final impact on olive trees, below an acceptable threshold.

The paper is structured as follows. We firstly present the model for the *Xylella fastidiosa* transmission by the adult meadow spittlebugs in two different scenarios: in greenhouse/nursery environments or in open field. Given a proper egg and juvenile control to reduce the overall population, we study the effectiveness of adult control actions through the analysis of the way in which the transmission depends on (1) spacing among trees; (2) number, frequency, intensity and kind of control actions; (3) vector mobility, adopting the worst case hypothesis. This conservative approach is abandoned in the Sensitivity Analysis section, in which we consider less restrictive values for the efficacy of the control actions and of other relevant parameters for the pathogen invasion. In the Discussion section, limits and perspective of our model are discussed. Details on the algorithm structure, parameters choice and simulations are given in the Method section.

## Results

In this paper, we construct a lattice model to simulate the bacterium/vector/tree infection interplay under different adult control actions. As suggested by Purcell^28^, in order to quantify the probability of a plant to be infected with *Xylella fastidiosa*, we consider four factors: vector infectivity, transmission efficiency, number of vectors and time they spent on the host.

Our aim is to exploit to what extent the infection can be mitigated even in un-favourable conditions. For this reason, we first focus on the worst case scenario, defined by the following assumptions: the control action efficacies assume relatively low values in respect to those actually achievable; the tree morphology is taken sparse, i.e. with few twigs per branch, corresponding to an higher number of vectors per twig; the vector feeding time is taken below the realistic value. Furthermore, the tree susceptibility and the local vector susceptibility to the bacterium acquisition are fixed to 100% for the three species of vectors.

### Model

In details, the model describing the pathogen transmission by the adult meadow spittlebugs is structured as follows.

- **The Orchard**. The orchard is projected in the plane and depicted as a simple square lattice. Olive trees and herbaceous vegetation are distributed on the lattice sites in order to mimic the typical structure of olive orchards, i.e. trees planted on parallel rows, at a distance *d*, with their canopies occupying the first and second nearest neighbour sites, and spontaneous herbs growing around the canopies. Each site of the lattice occupied by a tree represents a main branch of the tree, with a fixed number of twigs.
- **The Vector**. Vectors are distributed at random on the lattice sites and introduced in time according to a Gaussian distribution centred on forecasted vector eclosion time, in order to represent the circumstance that the transition from juvenile to adult instars is not simultaneous for the entire population, but takes place in a finite time window. They can move to nearest neighbour sites and, within each site occupied by an olive branch, from twig to twig. The vector dynamics is governed by different probabilities to perform different movements (from tree to tree, *p*_*tt*_, from herb to herb, *p*_*hh*_, from tree to herb, *p*_*th*_, and viceversa, *p*_*ht*_), which are fixed in order to mimic the insect seasonal preferences. In details, in late spring and early summer, olive sprouts are tender and spittelbugs typically prefer trees rather than vegetation. In late summer, when olive branches become hard, they are forced to move on vegetation in order to feed. Thus vectors are treated as particles moving at random on the lattice with a preferential choice for sites occupied by trees in the olive flowering time (${p}_{ht} > {p}_{th}$) and for herbaceous vegetation otherwise (${p}_{ht} < {p}_{th}$). Vectors do not feed on symptomatic infected trees. Adults surviving to the control actions die in winter, after eggs laying on herbs. The new brood originates the vector population of the following year.
- **The Transmission Process**. Infected adults transmit *Xylella fastidiosa* to olive trees with a fixed probability, while the acquisition of *Xylella fastidiosa* from infected trees is expressed as an appropriate function of the time occurred between the infection and the feeding act, to take in account the finite length of twigs and the finite time required by *Xylella fastidiosa* propagation in the plant vessels. A tree is classified as infected even if a single twig is infected.
- **The Vector Control**. The orchard is light tilled in winter as ovicidal control action; treatments against juveniles follow in April. The efficacy of early control actions versus eggs and juveniles are cautiously assumed to be significantly below a realistic threshold. Some simulations with more realistic values are discussed in Sensitivity Analysis section. The number of adults appearing at the eclosion time is determined by the efficacy of these preliminary treatments and fix the initial conditions for the adult transmission process. The orchard is subsequently treated with superimposed preventive and protective chemical control actions against adults.

The model presented falls under the category of SIR (Susceptible, Infected, Removed) model, with the susceptible (S) and the infected (I) being respectively the healthy and infected trees, while the removed correspond to symptomatic trees (which will die soon). In our disease restraint strategy, we do not consider the possibility to cut down infected trees, since in the present model symptomatic trees do not take part anymore to the epidemic spreading (i.e. in the SIR terminology, the symptomatic trees are Removed).

In Methods section we discuss in details the algorithm used to simulate the model and our choice for the model parameters (listed in Tables 1, 2 and 3).

**Table 1:** Orchard Parameters.

Spacing	6, 5, 4 m
Trees/ha	400
Length of twigs in tertiary branches	*L* = 15 cm
Number of twigs per tertiary branch	12, 36
Medium hardening time of twigs and standard deviation	185, 2 days
Medium time after infection of symptom appearance and standard deviation	730, 100 days

**Table 2:** Vector Parameters.

Stable population	1,000,000/ha
Offspring: eggs/female	Off = 400 − 100
Eggs to adult death rate, life table	*m* = 99.5% − 98%
Eggs control action efficacy	eff_*e*_ = 0.75 − 0.95
Juveniles control action efficacy	eff_*j*_ = 0.75 − 0.95
Medium time to eclosion and standard deviation	120, 5 days
Probability to move from twig to twig	*p*_*tt*_ = 0.1 − 0.7
Probability to move from branch to branch	*p*_*bb*_ = 0.05 − 0.35
Probability to move from tree to herb	*p*_*th*_ = 0.005 (tender sprouts) *p*_*th*_ = 1 (hard sprouts)
Probability to move from herb to tree	*p*_*ht*_ = 1 (tender sprouts) *p*_*ht*_ = 0 (hard sprouts)

**Table 3:** Transmission and Preventive control Parameters.

Vector susceptibility	*S*_0_ = 100%
Tree susceptibility	*S*_*t*_ = 100%
*Xylella fastidiosa* propagation rate in xylem	*v* = 0.167 *cm*/*day*
Time of the first insecticide treatment	day = 105
Frequency of treatments	5, 7, 14 days

### Closed orchard

We first consider numerical experiments in a closed (i.e. isolated) system. In this case spittlebugs born and die within the same orchard: no individual comes in or out. One infected vector is artificially introduced for the infection activation. The isolated orchard may be useful to mimic what happens in a controlled isolated experiment as for instance in a greenhouse, a plant nursery or a multiplication facility.

In the perspective of the worst possible case, we choose the efficacies of the egg and juvenile treatments equal to 75%, well below those realistically achievable nowadays, as experimented by one of the authors (Porcelli) during the very first vector control experiment given under the management of the *Xylella* invasion emergency by the Italian “Dipartimento della Protezione Civile”^27,29^ (see Sensitivity Analysis section for a discussion on how our findings depend on this choice). First we consider the case with no treatment against adults. Then we analyse the epidemic spreading by applying an increasing number of treatments with a fixed schedule. The treatments against adults consist in insecticides applied via spray or injection, with spray more effective than injection (for details on how the insecticide action is implemented in the model see Methods section). Different experiments with different numbers, frequencies and types of treatments are performed. The effects of the orchard structure, and the vector mobility are also analysed.

Simulations had run for a 10 years period: however, it emerges that the infection reaches an asymptotic state typically in 2 years (3 years in the case of lower mobility). This is a consequence of the fact that, as soon as trees manifest symptoms, they become unappealing for the insects, thus ceasing to be source of infection. Since in our model symptoms appear roughly after 2 years from infection and the most of infections occur during the first year, the epidemic spreading will be exhausted in roughly 3 years.

**Figure 2:**
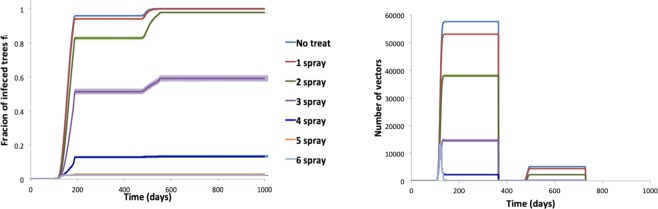
(**a**) Time evolution of *f*_*i*_ under different numbers of weekly treatments (sprayed insecticide, closed system, $d=4$, ${p}_{tt}=0.7$). (**b**) Corresponding time evolution of the total number of adults.

In Fig. 2, we plot the time evolution of the infection and of the adult number in the case of high vector mobility, intensive orchard and weekly spray treatments. We indicate with *f*_*i*_ the fraction of infected trees in the orchard. Due to the cyclic hardening of the trees after the flowering, although a finite number of adults is still present in the orchard, an arrested phase of the epidemic spreading is observed during each year. As shown in Fig. 2a, for the first two experiments (with no treatments and with only 1 treatment against adults) the asymptotic states correspond to totally infected orchards. We have verified (data not shown) that the first two treatments, although not enough effective to reduce the final state of infection, are fundamental to insure the efficacy of the following ones. Indeed if the first treatment against adults is delayed with respect to the eclosion time, all the other treatments are completely ineffective for the transmission control, although they can lead to the annihilation of the adult population.

Increasing the number of treatments, the number of adults (Fig. 2b) and, consequentially the asymptotic fraction of infected trees, gradually decrease, until further treatments become ineffective and then only detrimental for the environment. In the case studied in Fig. 2, we find that 5 weekly treatments are enough to reduce the asymptotic fraction of infective trees below the 3%.

**Figure 3:**
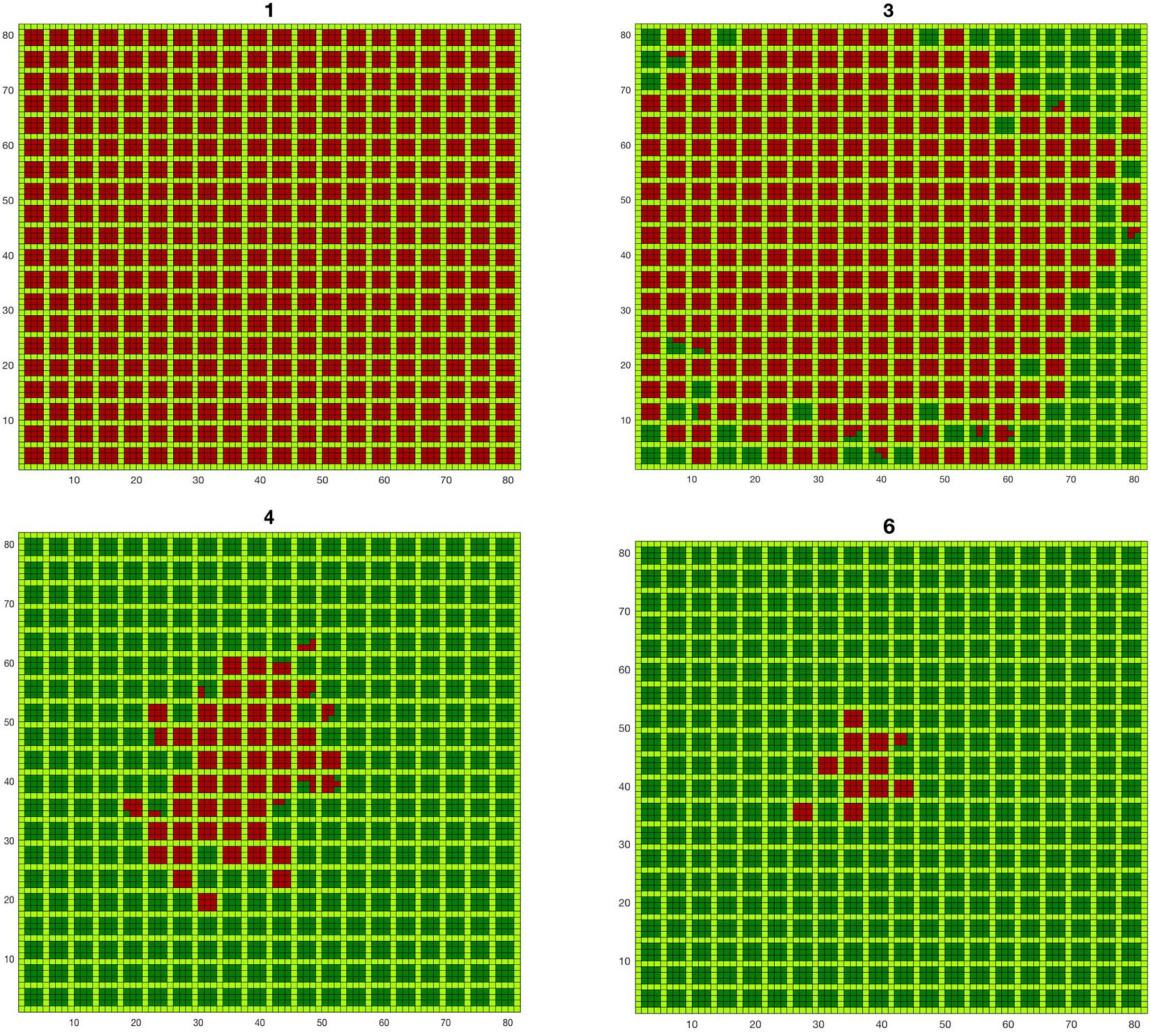
Orchard view of the epidemic spreading for different numbers of treatments (1, 3, 4 and 6) with herbs in light green, healthy trees in dark green and infected trees in red (sprayed insecticide, closed system, $d=4$, ${p}_{tt}=0.7$).

The orchard view of the epidemic spreading for a different number of treatments is plotted in Fig. 3. It shows that there are a few treatments that are decisive for the final impact of the infection (the treatments number 3–6 in the example are the ones that change the fate of the orchard). Below that number of treatments, the infection propagates to the whole orchard; above that set of treatments, the number of infected trees can only be marginally further reduced.

**Figure 4:**
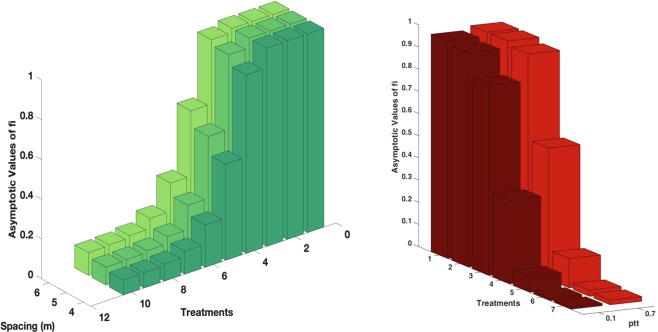
(**a**) Role of spacing - Asymptotic values of *f*_*i*_ with trees spacing $d=4$, 5, 6 (closed system, injected insecticide, ${p}_{tt}=0.7$). (**b**) Role of vector mobility - Asymptotic values of *f*_*i*_ with different vector mobility, low ${p}_{tt}=0.1$ and high ${p}_{tt}=0.7$ (closed system $d=4$, sprayed insecticide).

In order to analyse how these findings depend on the orchard structure, we change the distance between neighbour trees, keeping constant the vector density (i.e. the number of vector/ha). In Fig. 4a we see that intensive orchards, with smaller distance between neighbour trees (*d* = 4), are favoured with respect to the sparse ones (*d* = 6). Spacing could in principle also work in the opposite way: increasing the distance among trees could have the effect of slowing down the transmission process, by increasing the number of movements necessary for the vector to move from one tree to the other. However, our findings suggest that this effect is negligible and the number of vector/tree is the most relevant parameter controlling the transmission. Indeed, being the vector density (i.e. number of vector/ha) independent of the tree density (i.e. number of trees/ha), higher distances among trees entails an higher number of vector/tree, resulting in an increased risk of each tree to get infected. The fact that the parameter controlling the spreading of the disease is the mean number of insects per tree, is actually well-known in the mathematical epidemiology of vectored diseases in humans and animals^30,31^. If we instead consider an experiment, in which the tree density is increased, while keeping constant the number of insects per tree (data not shown), we observe a growth of the infection prevalence, due to the decreasing tree distance. Interestingly, if the distance is decreased of roughly 33% (from distance 6 to distance 4), in order to observe the same infection prevalence, the number of insects per tree has to be decreased of the same quantity. We intend to further analyse this point in a future work.

Actually data on vector mobility are lacking, and in our model the parameters regulating it (see Table 2) are arbitrarily chosen. In order to analyse the dependence of our findings on these parameters, in Fig. 4b we plot the asymptotic fraction of infected trees with a different number of treatments and two different values of the vector mobility parameter. As expected, the transmission process is slowed down if one assumes a reduced mobility. The effect is particularly appreciable for intermediate numbers of treatments. In order to improve the modelization, it would be useful to project and perform experiments aimed at collecting data on the vector mobility under in-field conditions.

**Figure 5:**
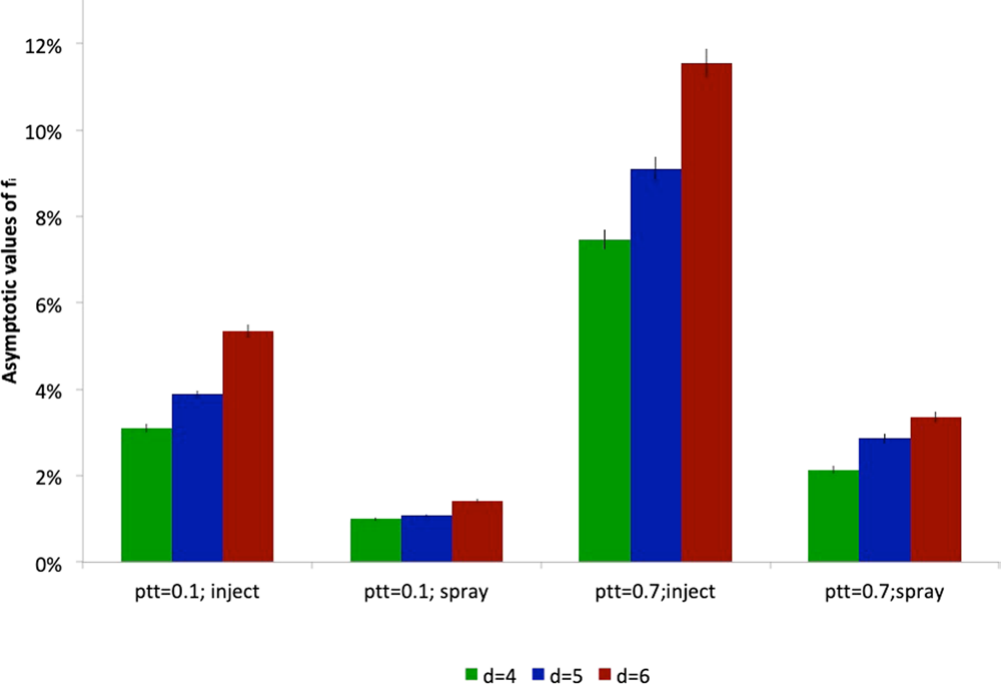
Asymptotic value of *f*_*i*_ with the maximum efficient number of treatments in the closed system.

Whatever is the vector mobility, structure of the orchard and type of treatment, we see that a sequence of an appropriate number of treatments with an appropriate schedule allows to reduce the impact of the disease below an acceptable fixed fraction of infected trees. For instance, the worst case of Fig. 5 (*d* = 6 with injection) has an asymptotic value of infected trees of roughly 12%. We verify that this value can be further reduced (below the 10%) increasing the frequency of treatments up to 1 treatment every 5 days (data not shown).

### Open orchard

In the open system scenario the orchard is not isolated and the infection can propagate from nearest neighbour infected orchard. In particular, we are interested to simulate what happens in the Containment zone interposed between the Infected zone and the Buffer zone. Those zones are established by the Regional Phytosanitary Service following the indications of the European Commission (data on the spreading of the disease and identification of the zones in Apulia are reported at^32^). Differently from the closed system, in the open one there is an in-coming and an out-going flux of vectors. We assume that, through a certain edge, the in-coming and the out-going flux of infected insects are proportional. This assumption is motivated by the hypothesis that the three areas are simultaneously treated with the same IPM strategy, and ensures that the in-coming flux of infected insects is very low in the years following the first one.

**Figure 6:**
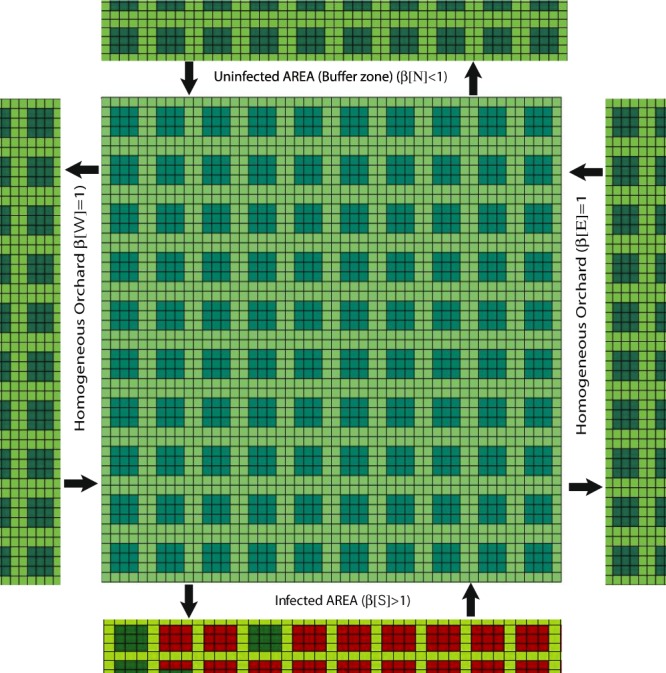
Schematic view of the open orchard (with herbs in light green, healthy trees in dark green and infected trees in red), with 4 distinct nearest neighbour orchards.

The orchard is supposed to be a square with one of the boundaries adjacent to an infected orchard (infected area), the opposite one adjacent to an uninfected one (buffer area), and the other two boundaries adjacent to orchards that are homogeneous on the infection point of view, as in Fig. 6. In order to simulate an infection that spreads in one direction, we assume, on different edges, different values for the probability of the in-coming and out-going vectors to be infected. We introduce a vector *β*[*i*], with *i* running over the 4 edges of the lattice (N,S,E,W), defined as

1 $$\beta [i]=\frac{{N}_{{\rm{\inf }}}^{{\rm{in}}}[i]}{{N}_{{\rm{\inf }}}^{{\rm{out}}}[i]}$$

with ${N}_{{\rm{\inf }}}^{{\rm{out}}/{\rm{in}}}[i]$ being the number of infected vectors of the out-going and in-coming flux through the boundary *i*, and choose $\beta [N] < 1$ (we choose 0.1) on the edge confining with the uninfected area, $\beta [S] > 1$ (we choose 2) on the edge confining with the infected one, and $\beta [E]=\beta [W]=1$ otherwise. We first consider these parameters constant in time, but they can be eventually regulated, year by year, in order to mimic the evolution of the infection in the adjacent orchards (see Sensitivity Analysis section). The assumption of a proportionality relation between the infected vectors in the in-coming and out-going flux through a certain edge, is motivated by the hypothesis that both infected and non infected area are simultaneously treated with the same IPM strategy. This assumption is not applicable in the present form to the case of neglected infected areas.

**Figure 7:**
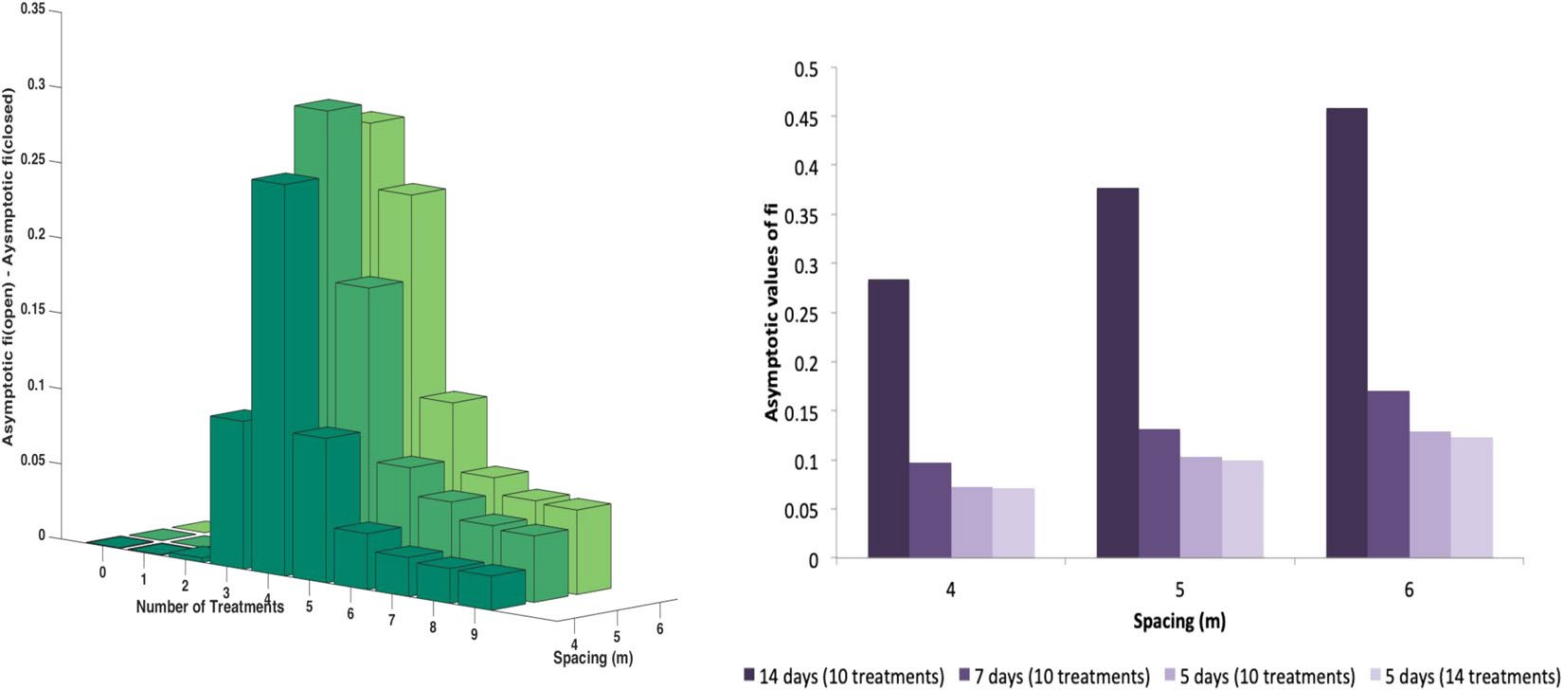
(**a**) Comparison between the open and closed system under different numbers of weakly treatments with injected insecticide (${p}_{tt}=0.7$). (**b**) Asymptotic values of *f*_*i*_ for different frequencies and number of treatments in the open system with $d=6$ and injected insecticide (${p}_{tt}=0.7$).

In all the cases considered, the open system results to be more susceptible to the invasion of the bacterium with respect to the closed one (see left panel of Fig. 7, where the closed and open systems are compared). The main differences between the open and closed system appear for intermediate number of treatments. However, increasing the frequency of the treatments, the asymptotic fraction of infected trees may be reduced to the closed case values, as shown in Fig. 7b.

## Sensitivity Analysis

In the previous section we have shown that, even in the worst case scenario, it is always possible, with an appropriate number, type and schedule of treatments, to reduce the impact of the infection below a certain threshold. On the other hand, the objective of minimising the number of treatments, in order to reduce the impact on the environment, should be always taken into account. Fortunately, relaxing the worst case hypothesis, one can reduce indeed the number of chemical treatments against adults, looking for the least impact on the environment.

**Figure 8:**
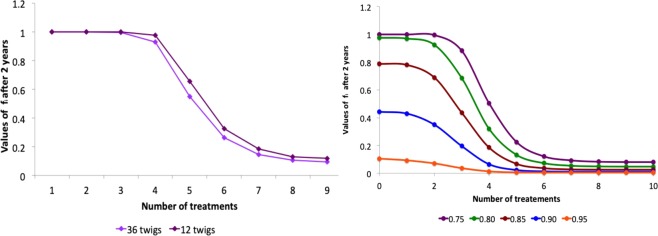
(**a**) Role of the number of twigs per branch - Asymptotic values of *f*_*i*_ as a function of the number of treatments on the adults (closed system, injected insecticide, *d* = 6). (**b**) Role of the efficacy of egg and juvenile control actions - Asymptotic values of *f*_*i*_ as a function of the number of treatments on the adults with egg and juvenile efficacies 0.75, 0.80, 0.85, 0.90 (closed system, injected insecticide, *d* = 6).

One of the worst case hypothesis adopted in the previous analysis was the assumption that the twigs on the branches are sparse. Increasing the number of twigs per branch, one has a reduction of the impact of the infection, as shown in Fig. 8a. Indeed, amping up the twigs per branch, the mean time, spent by the vectors on the same tree, also increases, resulting in a slowing-down of the infection, as can be proved by comparing the time evolution of the functions *f*_*i*_(*t*) in the case of 36 and 12 twigs per branch respectively (data not shown). The same effect can be obtained by acting directly on the value of the parameter representing the mean feeding time in the simulations.

Another conservative assumption adopted in the previous section concerns the values fixed for the efficacy of egg and juvenile treatments. Both can be realistically increased up to the 90–95%. In Fig. 8b one can see that the number of treatments against adults required to reduce the impact of the disease below the 10%, even in the case of sparse orchard, with high mobility and injection treatments, can be reduced significantly if the treatment on the eggs and juveniles are more effective.

It is also interesting to analyse how our findings in the open system depend on our choice of the parameters *β*[*i*]. In particular, comparing the simulations, with $\beta [S]=2$ on the edge confining with the infected orchard, to the simulations, with $\beta [S]=10$, we find that increasing *β*[*S*] slightly influences the infection. This is essentially due to the assumption that treatments are performed in confining orchards with the same timing and frequency, reducing in this way the infectivity potential of infected nearest orchards.

Data on the expansion of the *Xylella fastidiosa* in Apulia, show an advancement of the affected area of 20 km per year. As a consequence, the orchard that lies in the Buffer area, at a certain year, is likely to be raised to the Containment or to the Infected area in the following year. In order to represent this situation we run a set of simulations, in which we modify the value of the *β* parameters year by year. In particular, due to the propagation of infection to the previously non infected area, we increase the parameter *β*[*N*] on the edge confining with it, and due to the appearance of symptoms in the previously infected but non symptomatic one, we decrease the parameter *β*[*S*] on the opposite edge. We find that the relative error made by neglecting the time variability of *β*[*i*] is always less than 1% and well below this threshold in many cases.

## Discussion

**Figure 9:**
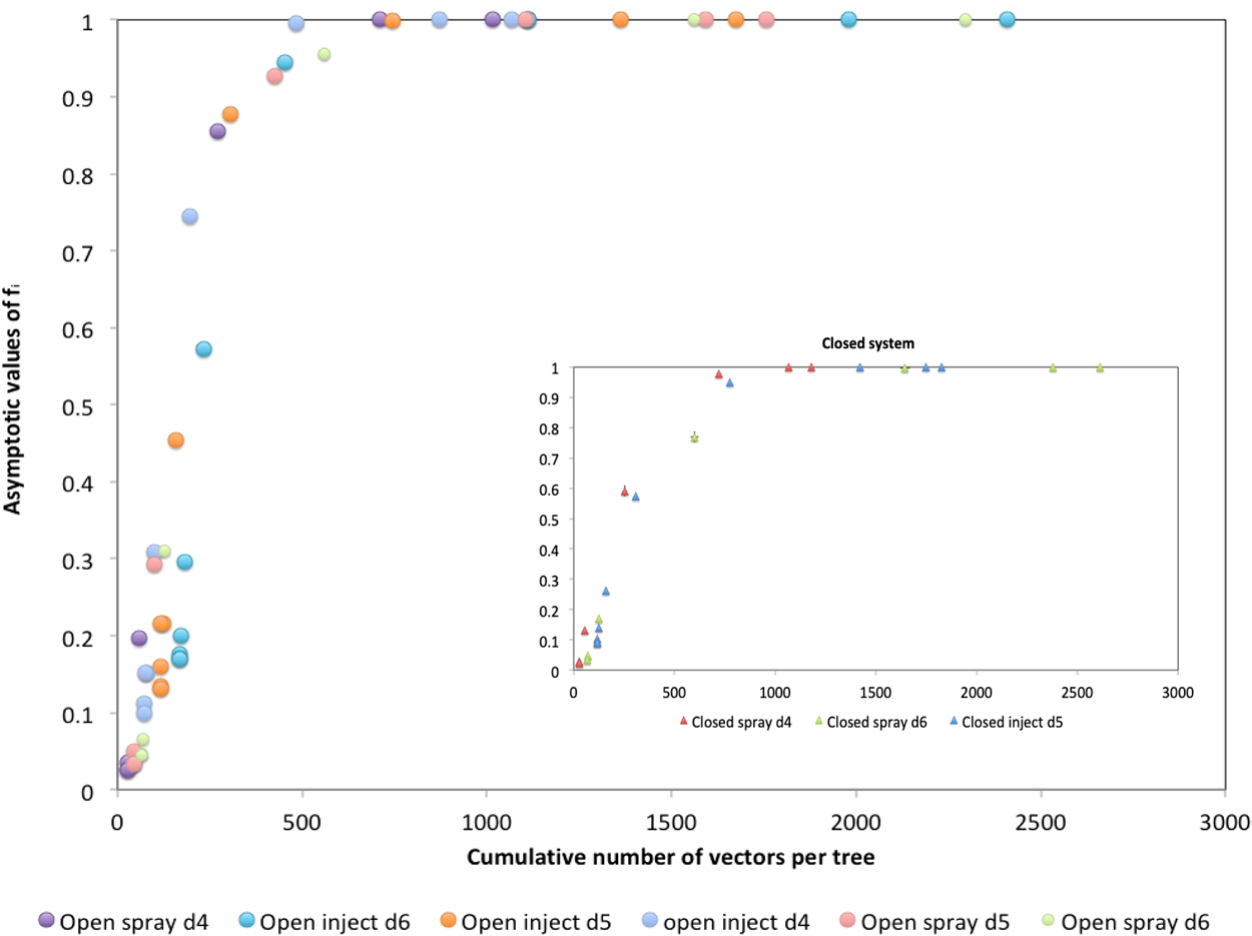
Asymptotic fractions of infected trees after 2 years plotted as a function of the cumulative number of vectors per tree. Main figure: open system. Inset: closed system.

In the framework of an Integrated Pest Management strategy, the model here presented shows that the hitherto unstoppable *Xylella fastidiosa pauca* ST53 invasion instead could be reversed. Our findings prove that the number of vector/tree is the most relevant parameter controlling the infection. In particular, this circumstance is evident in Fig. 9, where the asymptotic fractions of infected trees after 2 years are plotted as a function of the cumulative number of vectors per tree, in open and closed orchards. As we see, although we consider different situations (sprayed and injected insecticides, small and large spacings), the data collapse onto a single master curve, depending only on the conditions on the boundary of the orchard (closed or open). A transition is observed, from a state of low infectivity to a state of high infectivity, controlled by the cumulative number of vector/tree. Reducing this number below a certain threshold allows to hold the *Xylella* invasion within an acceptable range.

It is natural to wonder how much the model presented in this paper is realistic. Actually, in order to reproduce the vector dynamics and the resulting *Xylella fastidiosa* transmission on an olive orchard, the evaluation of the model parameters is crucial. While in some cases the literature comes to our aid (see for example the estimation of the number of eggs for female or the adult vector population discussed in Methods section), in other cases data are lacking, and the parameters are arbitrarily chosen (see for example the case of the vector mobility discussed in Results section). In all the unreliable cases, we choose to fix the parameters at the worst values on the point of view of the infection control, with the idea that, if the infection is controlled in the worst case, even more so, it can be controlled in the other ones. Data on the adults habits (time spent on the host, frequency of movements from one twig to another, etc.) could improve the realism of our modelization. Considering a three-dimensional model would also go in the direction of a more realistic description. In this case we would expect an increase of the mean time spent by the vectors on the same tree, and, consequentially, a slowing-down of the infection with respect to the two-dimensional case.

In first approximation, we do not take care of the fluctuations between different plants, fixing the number of branches per tree and twigs per branch, and analysing in this way only the mean behaviour of the orchard. Considering a more realistic distribution of twigs and branches around the mean value would allow to take into account such fluctuations. However we expect that the mean behaviour should reproduce the present findings.

In our model, we neglect the possibility of vectors to fly. We indeed assume that they move essentially by crawling or jumping, and allow only movements from a site to a nearest neighbour one, or smaller. Field measurements have shown that an adult instead may move for about 100 meters per day^33^, so an individual in its lifetime may travel up to hundreds or thousands of meters, particularly if aided by wind, even if with a very low probability. In future, we intend to modify our model in order to take into account such long distance movements.

Evidences of differential olive cultivar susceptibility to the bacterial infection and lesser vector efficiency in acquisition and transmission were obtained from phenotypic characterisation and molecular investigations. A higher bacterial population (up to 100 times) was consistently detected in olive trees of the most susceptible cultivars (i.e. Ogliarola and Cellina di Nardò, showing severe symptoms), compared to the trees of the FS17 clone (AKA Favolosa) and of the Leccino clones, showing milder symptoms and erratic distribution of the bacterium within the canopy^34–37^. In our model the tree susceptibility is always fixed as in the most susceptible cultivars. In future, we propose to investigate how our findings change considering less susceptible cultivars, or orchards with plants belonging to cultivars with different susceptibilities.

It is legitimate to wonder about the environmental impact of the chemical actions proposed within our model. To this purpose, we should remark that the olive tree is an anemophilous plant, i.e. pollen grains are wind-borne, and thus pollinators do not collect from olive flowers and are not endangered by the insecticide treatment. One could also wonder about the usage of potential biological control through adult-hunting natural enemies (e.g. carabids, spiders, mites, syrphids, coccinellids, birds and bats). Unfortunately few data exist about *Philaenus spumarius*, *Philaenus italosignus* and *Neophilaenus campestris* guild of antagonists, and a deep investigation is needed in order to sketch an IPM strategy in organic farm management. Indeed, the integration of natural enemies against adults in inundative control action could be useful to lower adult pre-reproductive population and the next brood. Therefore some efforts should be done in order to discover a predator community of the vector.

Question arises about the risk for resistance appearance to the insecticide use. On this point we should stress that Hemiptera Aphrophoridae vector population is mostly targeted by physical control means during the juvenile stage, to reduce the overall population size consistently. Adults that are vagrant will die because of the chemical control, in case they will feed on olive tree, and in a percentage depending on the formulate and distribution technique. The chemical action wishes to avoid infections (the first transmission) not to kill the more significant part of the vector population as an adult. The alternation of diverse chemical and physical control means on different phases of vector’s life cycles, as well as the univoltine biology of the vector, discourage the onset of an unwanted resistance. We conclude observing that, as all the strategies, our proposal need quantitative sampling *ex ante* - to program the control actions intensity and timing - and *ex post* - to measure their efficacy. Given that the suggested control actions are key-components of olive orchard IPM strategy, in case of consistent (orders of magnitude) reduction of vector population, we will recommend reducing consequently the intensity of the control actions to minimise the side effects. In conventional IPM farm management, this will result in the gradual lowering of the intensity, first, of physical control means, and last, of chemical ones, being the protective anti-infection chemical control a key point to protect plants from infection. We may further expect that reservoir (infected) plants will die because diseased and that the inoculum in the field will lower, giving rise to an equilibrium with a minimum and manageable number of new infections.

From this study it clearly emerges the distinction between the mere vector management, intended as the management of the vector population size (on eggs, juveniles and adults), and the transmission management, intended as the possibility to minimise the transmission events by adults survived to the control actions against juveniles. One could be erroneously led to think that the treatments against adults (i.e. vector adult management) coincide with the transmission management. Actually, adult treatments do not necessarily translate in an efficient transmission control, if their timing is not appropriate. Solutions with the entire adult population killed at the end of the first year and a completely infected orchard are still possible. Indeed, if the first treatment against adults is delayed with respect to their earlier appearance, all the other treatments are completely ineffective for the transmission control, although they eventually lead to the annihilation of the adult population. In this respect, the implementing 13 febbraio 2018 decree^38^, and successive regulations, fix the first treatment against adults at the beginning of May, followed by other treatments with a frequency of 14 days, and aim at the vector rather than transmission control. According to our model, such a prescription remains completely ineffective. Indeed it facilitates the *Xylella fastidiosa* invasion either for the wrong timing, that delays the action in respect to the adult appearance, or for the low frequency of the treatments, that permits transmissions of survived vectors. Furthermore, the delayed symptoms of infection make the *Xylella fastidiosa* invasion management particularly insidious because the infected area precedes of many kilometres the symptomatic ones. However, the search for *Xylella fastidiosa* infection, to eventually declare the area as infected, are symptom dependent and the state of alert is given too late in time and far from the invasion line to be useful, consequently. It was precisely the absence of prompt evidence of infections that induced the policymakers to wrongly adopt differentiated treatments among confinement and infected areas^38^. The latter is another point of weakness in this decree. On the contrary, the front of the vector control actions should precede those of symptoms to have a chance to arrest the infection. The right suggestion is thus that infected and containment areas should undergo the same IPM strategy and vector control pressure, as in our open orchard simulations, to have a chance to stop the *Xylella fastidiosa* invasion over the country.

## Methods

The properties of the model are studied using numerical simulations. We perform 100 independent realisations of each experiment with different random generators. The simulated data, and their errors, are evaluated respectively as mean values and standard deviations over the independent processes. Although the data are in general plotted for a smaller period, the simulations are done for an interval of 10 years.

The code is developed in analogy with Monte Carlo simulations of physics models. The main difference is that here particles are replaced by insects, then the dynamics is not regulated by a Hamiltonian, but by biological constraints.

The simulation codes, formulated in C-language, are available in the Supplementary Information.

Each tree occupies 9 sites of the 2*d* lattice (the centre and all its nearest and second-nearest neighbours), sites corresponding to tertiary branches of plants, with *N*_*twig*_ twigs per branch. Trees are characterised by an hardening time, ${\tau }_{hard}$: i.e. tree becomes hard at ${\tau }_{hard}$ and becomes tender again in spring. Infected trees are also characterised by a symptom appearance time, ${\tau }_{sym}$. Both times are extracted for each plant from Gaussian distributions, and fixed for the entire simulation. The health state of twigs and the time of infections are acquired every time step.

In nature eclosion does not start exactly in the same day. In order to take in account this variability, at the beginning of each year, we randomly chose the eclosion time from the time interval $[{\tau }_{ecl}-3\,{\rm{days}},{\tau }_{ecl}+3\,{\rm{days}}]$ (where ${\tau }_{ecl}$ is put equal to 120 day). Year by year, insects are distributed at random on the lattice sites and introduced in time according to a Gaussian distribution centred on the mean eclosion time of that specific year. The total number of insects, which appear after eclosion at year *y* are evaluated as

2 $${A}^{y}=\frac{{N}_{{\rm{v}}}^{y-1}}{2}\times {\rm{Off}}\times (1-m)\times (1-{{\rm{eff}}}_{e})\times (1-{{\rm{eff}}}_{j})$$

where ${N}_{{\rm{v}}}^{y-1}$ is the number of adults surviving at the end of the year *y* − 1, Off is the number of eggs per female, *m* is the eggs to adult death rate, and eff_*e*_ and eff_*j*_ are the efficacy of egg and juvenile control actions, respectively. The eggs to adult death rate m varies as a function of the number of eggs per female, Off. Opinions on the total laid eggs per female are quite diverse, varying from a minimum of 10^39^ through intermediate 18–51 per female^40–42^ to a maximum of 350–400^43^. Diverse estimated number of eggs may depend on environmentally-driven female variable fertility. We choose the maximum of 400 eggs per female assigning an egg mortality m = 99.5%, corresponding to a pair of survivors every 400 eggs, which ensures to have a stable population (or equivalently 100 eggs per female with a mortality m = 98%).

In winter all the insects, survived to treatments against adults, die. The health state and the position of insects are acquired every time step.

In details, the structure of the code is the following. The main routine calls the other ones, respectively for

1. memory allocation;
2. definition of lattice structure;
3. placement of olive trees and herbs on the lattice;
4. regulation of birth, death and dynamics of insects, and, consequentially, spreading of the disease.

**Figure 10:**
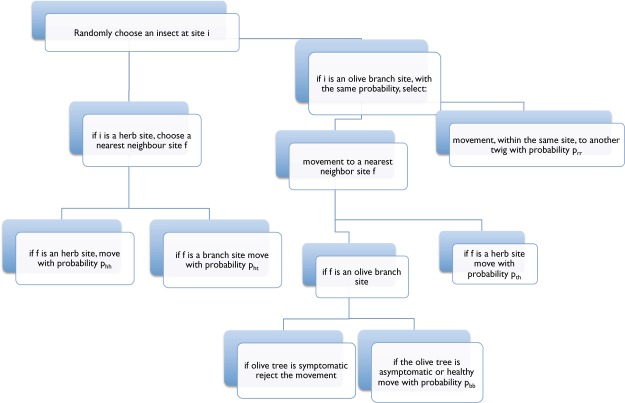
Algorithm - Dynamics.

The heart of simulation code is given by insect dynamics. The routine, which regulates the dynamics, is called once daily. In one day, each insect tries to move every half hour (i.e. 48 times), so 30 minutes is our time step (which corresponds to the minimum feeding time for the insects). In details, for the *j*–th insect located at site *i* (see Fig. 10):

1. If the site *i* is occupied by herb, a nearest neighbour destination site *f* is randomly chosen:
  if *f* is occupied by herb, individual moves with probability *p*_*hh*_;
  if *f* is occupied by a branch of an olive tree, individual moves with probability *p*_*ht*_.
    If the site *i* is occupied by a branch of an olive tree, the individual tries to move with equal probability to another twig or to the nearest neighbour destination site, *f*, randomly chosen on the lattice:
  movements to twigs of the same branch are accepted with probability *p*_*tt*_;
  movements to a different branch are accepted with probability *p*_*bb*_;
  movements to herb with probability *p*_*th*_.
  The values of the probabilities, listed in Table 1, depend on the specific period of the year and on the symptomatic status of infected tree: movements to symptomatic and hard trees are always rejected. We assume *p*_*bb*_ to be half of *p*_*tt*_. This choice is motivated by the morphological structure of branches and twigs.
2. After each movement, we control the state of health of plants and insects:
  healthy plants contract the infection from infected insects with susceptibility *S*_*t*_;
  healthy insects acquire the bacterium with a transmission probability that depends on the time since infection (in order to take into account that twigs have finite length, but the infection process is point-like and bacteria propagation process into plant vessels requires a finite time). Assuming a bacteria propagation velocity of *v* = 5 cm/month and a twig length of roughly *L* = 15 cm, after 3 months from infection, the whole twig will be infected and the susceptibility will be equal to *S*_0_. Thus, we assume the infection probability of a vector feeding at time *t* on a twig infected at time *t*_*inf*_ to be
  3 $${S}_{{\rm{v}}}=\{\begin{array}{ll}{S}_{0}(t-{t}_{{\inf }})\frac{v}{L} & (t-{t}_{{\inf }}) < 3\,{\rm{months}},\\ {S}_{0} & {\rm{otherwise}}.\end{array}$$
3. In presence of treatments against adults, after each bite the insect has a probability to die that depends on the time occurred since each treatment. In particular, we fix it equal to

4 $${P}_{d}={P}_{0}\,\sum _{i=1}^{{N}_{{\rm{treat}}}}{e}^{-(t-{t}_{i})/\tau },$$

where *P*_0_ and $\tau$ are two numerical constants depending on the specific insecticide and its distribution procedure, *t*_*i*_ is the time in which the *i*-th treatment is done, and *t* is the current time. *P*_0_ and $\tau$ are chosen in such a way to reproduce the cumulative efficacy of the reference insecticides (Cumulative Imidacloprid mortality at day 14 with spray distribution: 92.8%, Cumulative Imidacloprid mortality at day 14 with injection distribution: 67.1%) as in^26^. The time of the first insecticide treatment is fixed to day 105, which corresponds to the earlier appeareance of adults. We neglect any natural mortality factor on the adult population, except that induced by the pest management actions.

We repeat the above steps *N*_v_ times, where *N*_v_ is the current number of insects. The number of infected tertiary branches are acquired every step.

Clearly, vector population size estimation is compulsory in IPM planning, and in *ex ante* tuning of control action intensity and *ex post* evaluation of control action efficacy. The biomimetic sampling method called AquaSamPling^44^ estimates a juvenile population ranging from less than 100,000 to 30,000,000 of individuals per hectare. A recent paper^45^ suggests a population level of about 500,000 vectors per hectare. For our purpose, we choose an initial population size ${N}_{{\rm{v}}}^{0}=\mathrm{1,000,000}$, since this is the worst case for the most common over the country.

Wishing the worst case in acquisition/transmission process we will overlook the knowledge in acquisition/transmission rate given in references^9,11,12^ considering all (*S*_0_ = 100%) the Aphrophoridae guild adults able to locally acquire almost instantaneously after eclosion at first feeding on infected twig, and all (*S*_*t*_ = 100%) the guild to transmit instantaneously during the second and all subsequent feedings on olive trees.

The model parameters are listed in Tables 1, 2 and 3.

## Supplementary information

Supplementary Information
