# Supplementary material for: A lattice model to manage the vector and the infection of the Xylella fastidiosa on olive trees
Source: Sci Rep. 2019 Jun 19;9:8723. doi: 10.1038/s41598-019-44997-4 (PMC6584701; doi:10.1038/s41598-019-44997-4)
Supplement: Supplementary file 1 — Supplementary Information [file 41598_2019_44997_MOESM1_ESM.pdf]

Supplementary Information

A lattice model to manage the vector and the infection of the *Xylella fastidiosa* on olive trees

Annalisa Fierro, Antonella Liccardo, Francesco Porcelli

The present supplementary information contains the computer codes used for numerical simulations of the Closed and Open Scenario discussed in the article. The codes are written in C-language.

1. Closed System

```
#include <stdlib.h>
#include <stdio.h>
#include <math.h>
#define NRANSI

// CLOSED SYSTEM

// PARAMETERS ***********************

// euclidean dimension of the lattice
#define d 2
// lattice size
 #define L 81

// spacing among trees
#define spaz 4

// duration of simulation
#define ST 200

// probability of insect to become infect pp*(nstep-tinf)
#define pp 0.0111

// probability of an olive tree to become infect
#define pm 1.0

// initial efficacy of the insecticide
// 0.0275 spray
// 0.011 injection
#define pd 0.0275
// decay time of the insecticide
#define tau 3.

// initial number of infected insects
#define Ninf 1

// number of twigs per branch
#define Ntwig 12

// number of treatments against adults
```

```
#define Ninterv 0
// time of the first insecticide treatment
#define t0 105
// frequency of treatments
#define delta 7

// probability to move from twig to twig
#define ptt 0.7

// probability to move from branch to branch
#define pbb 0.35

// probability to move from herb to herb
#define phh 0.005

// medium time to eclosion
#define tecl 120.0
// standard deviation of the time to eclosion
#define sigmas 5

// medium hardening time of twigs and branches
 #define tind 185.0
// standard deviation of the hardening time
#define sigmad 2.0

// medium time after infection of symptom appearence
#define tsint 730.0
// standard deviation of symptom appearence time
#define sigma 100

#define PIG 3.14159265358979323846

//VARIABLES*****************************************

static int mype;
static int h,max,Ntree;
static int nstep,Nv,Ne,Ninf0,Newborn;
static int Ninsect;

static int **N,**NN;
static int *posix,*site;
static int *ill,*port;
static int **ill_twig,*twig;
static int *ti;

static double **diff;
static double **tinf,*tsym,*thar;

static void memory_allocation(void)
  {

// MEMORY ALLOCATION********************************

   int i,j;

// nearest neighbour matrix
   N=malloc(h*sizeof(int*));
// second nearest neighbour matrix
   NN=malloc(h*sizeof(int*));
   for (i=0;i<h;i++)
       {
        N[i]=malloc(max*sizeof(int));
        NN[i]=malloc(max*sizeof(int));
       }
```

```
// diffusion matrix
   diff=malloc(2*sizeof(double*));
   for (i=0;i<2;i++) diff[i]=malloc(2*sizeof(double));


// 0 if the site i-th is occupied by herb, and >0 otherwise
   site=malloc(max*sizeof(int));
// 1 if the site is occupied by an ill tree, 0 otherwise
   ill=malloc(max*sizeof(int));
// tree in site i-th becomes symtomatic at a time tsym[i] after infection
   tsym=malloc(max*sizeof(double));
// tree in site i-th becomes hard at a time thar[i]
   thar=malloc(max*sizeof(double));


// 0 or 1 if the twig i-th in the site j-th is healthy or not
   ill_twig=malloc((Ntwig+1)*sizeof(int*));
   for (i=0;i<=Ntwig;i++) ill_twig[i]=malloc(max*sizeof(int));

// infection time of twig i-th in the site j-th
   tinf=malloc((Ntwig+1)*sizeof(double*));
   for (i=0;i<=Ntwig;i++) tinf[i]=malloc(max*sizeof(double));


// position of the k-th insect
   posix=malloc((Ninsect+1)*sizeof(int));

// twig of the branch in the site posix[j], where the j-th insect is on
   twig=malloc((Ninsect+1)*sizeof(int));

// health state of the j-th insect (0 healthy, 1 ill)
   port=malloc((Ninsect+1)*sizeof(int));

   for (i=0;i<max;i++)
        {
         site[i]=ill[i]=tsym[i]=thar[i]=0;
         for (j=1;j<=Ntwig;j++) tinf[j][i]=ill_twig[j][i]=0;
        }

   for (j=1;j<=Ninsect;j++)
        {
         posix[j]=port[j]=0;
         twig[j]=-10;
        }
// time of the j-th insecticide treatments
   ti=malloc((Ninterv+1)*sizeof(int));
   ti[1]=t0;
   for (j=2;j<=Ninterv;j++) ti[j]=ti[j-1]+delta;
  }
static void init_next(void)
  {
   int i,j;

// LATTICE STRUCTURE *******************************************

// definition of nearest neighbours and second-nearest neighbours
   for (j=0;j<max;j++)
     {
       N[0][j]=j-1;
       N[1][j]=j-L;
       N[2][j]=j+1;
       N[3][j]=j+L;
```

```
       NN[0][j]=j-1-L;
       NN[1][j]=j+1+L;
       NN[2][j]=j-1+L;
       NN[3][j]=j+1-L;
     }
  }
static void init(void)
  {

// PLACEMENT OF TREES ON THE LATTICE
*****************************************************************

   int i,j,ix,iy,s0,next;
   double x,p;

   Ne=max;
   Ntree=0;

// placement of olive tree center in the lattice site with abscissa and ordinate
ix%spaz=2 and iy%spaz=2
// each plant occupies all the nearest and the second-nearest neighbours of such
site

   for (i=0,s0=0;i<max;i++)
       {
// abscissa of the i-th site
         ix=i%L;
// ordinate of the i-th site
      iy=i/L;
        if ((ix%spaz==2)&&(iy%spaz==2))
           {
            s0++;
            site[i]=s0;
            Ne--;
            Ntree++;

// TREES ATTRIBUTES
******************************************************************************

// the time of symptom appearence, after infection, is randomly chosen from a
Gaussian distribution
            for (j=1;j<=10000000;j++)
                {
                 x=tsint+(double)(2*(int)(2.0*drand48())-1)*3.0*sigma*drand48();
                 p=exp(-(x-tsint)*(x-tsint)/(2.0*sigma*sigma));
                 if (drand48()<p)
                     {
                      tsym[i]=x;
                      break;
                     }
                }
// the hardening time is randomly chosen from a Gaussian distribution

            for (j=1;j<=10000000;j++)
                {
                 x=tind+(double)(2*(int)(2.0*drand48())-1)*3.0*sigmad*drand48();
                 if (x<0) x=0;
                 p=exp(-(x-tind)*(x-tind)/(2.0*sigmad*sigmad));
                 if (drand48()<p)
                     {
                      thar[i]=x;
                      break;
                     }
```

```
                 }
// tsym and thar are equal for each branch of the tree
            for(j=0;j<h;j++)
                {
                 next=N[j][i];
                 site[next]=s0;
                 Ne--;
                 tsym[next]=tsym[i];
                 thar[next]=thar[i];
                }
            for(j=0;j<h;j++)
                {
                 next=NN[j][i];
                 site[next]=s0;
                 Ne--;
                 tsym[next]=tsym[i];
                 thar[next]=thar[i];
                }

            }
        }
  }
void dynamics(void)
  {
   int i,j,k;
   int i0,j0,s0,s1,x,kh,next;
   int n1,n2,dead,u0,u1,ix,iy;
   double p,pcont;

   FILE *stream;
   char buffer[100];

// INTRODUCTION OF VECTORS ON THE LATTICE **************

// eclosion of Newborn insects at the current time nstep
//
   for (i=1;i<=Newborn;i++)
       {
        Nv++;
        x=(int)(max*drand48());
        posix[Nv]=x;
        if (site[x]>0)
          {
           j0=1+(int)(Ntwig*drand48());
           twig[Nv]=j0;
          }
        port[Nv]=0;

        if ((nstep<365)&&(Ninf0<Ninf))
           {
            Ninf0++;
            port[Nv]=1;
           }
       }

// VECTOR DYNAMICS AND DISEASE SPREADING
*****************************************************

   if (Nv>0) for (j=0;j<48;j++)
       {
        for (i0=1,dead=0;i0<=Nv;i0++)
            {
// x is the position of insect i0
             x=posix[i0];
```

```
              s0=site[x];
              if (s0>0) //i0 is on the olive tree s0
                 {
                  k=(int)(2.0*drand48());
//with probability 0.5 the vector i0 rests on the same branch and tries to move
to another twig
                  if (k==0)
                  {
// the vector i0 moves to another twig of the same branch
// posix does not change
// another twig is chosen

                  for (i=0;i<1000000;i++)
                     {
                      j0=1+(int)(Ntwig*drand48());
                      if (j0!=twig[i0]) break;
                     }

                  if (drand48()<ptt) twig[i0]=j0;
// the vector i0 moves from  twig[i0] to j0
                  else j0=twig[i0];
// the vector i0 does not move


// if the olive branch is tender, the vector eats
                  if ((nstep%365)<thar[x])
                      {
// if the twig is healthy and the vector is infected, the twig becomes infected
// with probability pm
                       if ((ill_twig[j0][x]==0)&&(port[i0]==1)&&(drand48()<pm))
                          {
                           ill[x]=ill_twig[j0][x]=1;

tinf[j0][x]=(double)nstep+(double)(i+1)/(double)(48*Nv);
                          }
// if the twig is infected and the vector is healthy, the vector becomes
infected with probability pcont
                       if ((ill_twig[j0][x]==1)&&(port[i0]==0))
                          {
                           pcont=pp*(nstep-tinf[j0][x]);
                           if (pcont>1) pcont=1;
                           if (drand48()<pcont) port[i0]=1;
                          }

                       p=0;
// the vector dies after a bite with probability p
                       for (i=1;i<=Ninterv;i++) if ((nstep%365)>=ti[i])
p=pd*exp(-(double)((nstep%365)-ti[i])/tau);

                       if (drand48()<p)
                           {
                            posix[i0]=-10;
                            port[i0]=0;
                            dead++;
                           }
                       }
                    }
                 else
// the vector i0 moves to a nearest neighbour site
                    {
// a nearest neighbour site is randomly chosen
                     ix=x%L;
                     iy=x/L;
```

```
                    if (((nstep%365)>=thar[x])&&(ix%spaz!=2)&&(iy%spaz!=2))
                       {
// the olive branch is no more tender
// a direction, which allows the insect to leave the plant, is randomly chosen

                        for(i=0;i<1000000;i++)
                           {
                            kh=(int)(h*drand48());
                            next=N[kh][x];
                            s1=site[next];
                            if (s1==0) break;
                           }
                       }

//if the olive branch is tender
                    else
                       {
                        kh=(int)(h*drand48());
                        next=N[kh][x];
                        s1=site[next];
                       }

                    u0=1;//the departure site is on an olive tree
                    if(s1>0) u1=1; //the arrival site is on an olive tree
                    else u1=0; //the arrival site is on herb

                    p=diff[u0][u1];

                    if (s1>0) //the arrival site is on an olive tree
                        {
// a twig is randomly chosen
                         j0=1+(int)(Ntwig*drand48());
// if the olive tree in the arrival site is symtomatic the movement is rejected
                         if (nstep>=(tinf[j0][next]+tsym[next])) p=0;
                        }
// if the olive tree in the departure site is hard the movement is accepted with
probability 1
                     if ((nstep%365)>=thar[x]) p=1;

                     if (drand48()<p)
                        {
                         posix[i0]=next;
                         if (s1>0) twig[i0]=j0;
                        }
                     else
                        {
                         j0=twig[i0];
                         next=x;
                        }
                     if ((s1>0)&&((nstep%365)<thar[next]))
                        {
// the arrival site is on a tender olive
                         if ((ill_twig[j0][next]==0)
                             &&(port[i0]==1)&&(drand48()<pm))
                            {
                             ill[next]=ill_twig[j0][next]=1;
                             tinf[j0][next]=(double)nstep+
                             (double)(i+1)/(double)(48*Nv);
                             }
                         if (ill_twig[j0][next]==1)
                            {
                             pcont=pp*(nstep-tinf[j0][next]);
                             if (pcont>1) pcont=1;
                             if((port[i0]==0)&&(drand48()<pcont)) port[i0]=1;
```

```
                                }
                          p=0;
                      for (i=1;i<=Ninterv;i++) if ((nstep%365)>=ti[i]) p=pd*exp(-
(double)((nstep%365)-ti[i])/tau);
                          if (drand48()<p)
                             {
                              posix[i0]=-10;
                              port[i0]=0;
                              dead++;
                             }
                          }
                      }
                  }
               if (s0==0)  //i0 is on herb
                  {
//a nearest neighbor site is randomly chosen

                  kh=(int)(h*drand48());
                  ix=x%L;
                  iy=x/L;
//since the lattice is closed, if the departure site is on the lattice edge some
directions
//are not possible
                  if ((ix==0)&&(iy==0))
                     for(i=0;i<1000000;i++)
                     {
                      if ((kh!=0)&&(kh!=1)) break;
                      kh=(int)(h*drand48());
                     }
                  else if ((ix==0)&&(iy==(L-1)))
                     for(i=0;i<1000000;i++)
                     {
                      if ((kh!=0)&&(kh!=3)) break;
                      kh=(int)(h*drand48());
                     }
                  else if ((ix==(L-1))&&(iy==0))
                     for(i=0;i<1000000;i++)
                     {
                      if ((kh!=1)&&(kh!=2)) break;
                      kh=(int)(h*drand48());
                     }
                  else if ((ix==(L-1))&&(iy==(L-1)))
                     for(i=0;i<1000000;i++)
                     {
                      if ((kh!=3)&&(kh!=2)) break;
                      kh=(int)(h*drand48());
                     }
                  else if (ix==0)
                     for(i=0;i<1000000;i++)
                     {
                      if (kh!=0) break;
                      kh=(int)(h*drand48());
                     }
                  else if (ix==(L-1))
                    for(i=0;i<1000000;i++)
                     {
                      if (kh!=2) break;
                      kh=(int)(h*drand48());
                     }
                  else if (iy==0)
                   for(i=0;i<1000000;i++)
                     {
                      if (kh!=1) break;
                      kh=(int)(h*drand48());
```

```
                    }
                 else if (iy==(L-1))
                   for(i=0;i<1000000;i++)
                    {
                     if (kh!=3) break;
                     kh=(int)(h*drand48());
                    }

                 next=N[kh][x];
                 s1=site[next];

                 u0=0; //the departure site is on herb
                 if(s1>0) u1=1; //the arrival site is on an olive tree
                 else u1=0; //the arrival site is on herb

                 p=diff[u0][u1];

                 if (s1>0) //the arrival site is on an olive tree
                     {
                      j0=1+(int)(Ntwig*drand48());
// if the olive branch in the arrival site is symtomatic the movement is
rejected
                      if (nstep>=(tinf[j0][next]+tsym[next])) p=0;
// if the olive branch in the arrival site is hard the movement is rejected
                      if ((nstep%365)>=thar[next]) p=0;
                     }

                if (drand48()<p)
                    {
                     posix[i0]=next;

                     if (s1>0) //the arrival site is on an olive tree
                        {
                         twig[i0]=j0;
                         if ((ill_twig[j0][next]==0)
                              &&(port[i0]==1)&&(drand48()<pm))
                            {
                             ill[next]=ill_twig[j0][next]=1;

tinf[j0][next]=(double)nstep+(double)(i+1)/(double)(48*Nv);
                            }

                         if (ill_twig[j0][next]==1)
                            {
                             pcont=pp*(nstep-tinf[j0][next]);
                             if (pcont>1) pcont=1;
                             if((port[i0]==0)&&(drand48()<pcont)) port[i0]=1;
                            }
                         p=0;
                         for (i=1;i<=Ninterv;i++) if ((nstep%365)>=ti[i])
p=pd*exp(-(double)((nstep%365)-ti[i])/tau);

                         if (drand48()<p)
                            {
                             posix[i0]=-10;
                             port[i0]=0;
                             dead++;
                            }
                        }
                    }
                }
              }
      if (dead>0)
          {
```

```
            for (n1=Nv;n1>0;n1--) if(posix[n1]<0) for (n2=n1+1;n2<=Nv;n2++)
                 {
                  posix[n2-1]=posix[n2];
                  port[n2-1]=port[n2];
                  twig[n2-1]=twig[n2];
                 }
            Nv=Nv-dead;
            for(n1=Nv+1;n1<=Nv+dead;n1++) port[n1]=0;
           }
       }

}

//********************* MAIN ********************************************

int main(argc,argv)
int argc;
char **argv;
  {
   long int seed;
   int i,infected;
   double p,tsfarf;

   FILE *stream;
   char buffer[100];

   seed=775;
   seed+=(long)mype;

   srand48(seed);
// total number of lattice site
   max=L*L;
// number of nearest neighbours
   h=2*d;
// number of olive trees
   Ntree=(L-1)*(L-1)/(spaz*spaz);

   Nv=Ninf0=0;
   Ninsect=57600;

   memory_allocation();
   init_next();
   init();

// diffusion matrix

   diff[0][0]=1.0;
   diff[1][1]=pbb;
   diff[0][1]=1.0;
   diff[1][0]=phh;

   for (nstep=0;nstep<ST;nstep++)
      {
       for (i=0,infected=0;i<max;i++) if ((site[i]>0)&&(ill[i]>0)) infected++;

       sprintf(buffer, "inf_%i_%lg_%i_%i_%i",L,ptt,spaz,Ninterv,mype);
       if (nstep==0) stream = fopen(buffer, "w");
       else stream = fopen(buffer, "a");

       fprintf(stream,"%i %lg\n",nstep,(double)infected/(double)(9*Ntree));

       fclose(stream);
```

```
       if ((nstep%365)==0)
           {
            if (nstep!=0) Ninsect=(int)(0.09*(double)Nv);

            Nv=0;
            for (i=1;i<=Ninsect;i++) port[i]=0;

// the mean time to eclosion changes randomly from tecl-3 to tecl+3
            tsfarf=tecl+(2.0*(int)(2.0*drand48())-1)*(int)(4*drand48());
           }

       p=(1.0/sqrt(2.0*PIG*sigmas*sigmas))*exp(-((double)(nstep%365)-
tsfarf)*((double)(nstep%365)-tsfarf)/(2.0*sigmas*sigmas));

       Newborn=(int)((double)Ninsect*p);
// number of newborn per day at time nstep

       dynamics();
      }
    return 0;
  }
```

2. Open System

```
#include <stdlib.h>
#include <stdio.h>
#include <math.h>
#define NRANSI

// OPEN SYSTEM

//PARAMETERS ************************


// euclidean dimension of the lattice
#define d 2
// lattice size
 #define L 81

// spacing among trees
#define spaz 4

// duration of simulation
#define ST 201

// probability of insect to become infect pp*(nstep-tinf)
#define pp 0.0111

// probability of an olive tree to become infect
#define pm 1.0

// initial efficacy of the insecticide
#define pd 0.0
//0.0275 spray
//0.011  injection
// decay time of the insecticide
#define tau 3.

// initial number of infected insects
```

```
#define Ninf 1

// number of twigs per branch
#define Ntwig 12

// number of treatments against adults
#define Ninterv 0
// time of the first insecticide treatment
#define t0 105
// frequency of treatments
#define delta 7

// probability to move from twig to twig
#define ptt 0.7

// probability to move from branch to branch
#define pbb 0.35

// probability to move from herb to herb
#define phh 0.005

// medium time to eclosion
#define tecl 120.0
// standard deviation of the time to eclosion
#define sigmas 5

// medium hardening time of twigs and branches
 #define tind 185.0
// standard deviation of the hardening time
#define sigmad 2.0

// medium time after infection of symptom appearence
#define tsint 730.0
// standard deviation of symptom appearence time
#define sigma 100

#define fact 1.0

#define PIG 3.14159265358979323846

//VARIABLES ****************************************

static int mype;
static int h,max,Ntree;
static int nstep,Nv,Ne,Ninf0,Newborn;
static int Ninsect;

static int **N,**NN;
static int *posix,*site;
static int *ill,*port;
static int **ill_twig,*twig;
static int *ti;

static double **diff;
static double **tinf,*tsym,*thar;

static double *finf;
static int out,*inf_in,*inf_out;

static void memory_allocation(void)
  {
// MEMORY ALLOCATION ********************************

   int i,j;
```

```
// nearest neighbour matrix
   N=malloc(h*sizeof(int*));
// second nearest neighbour matrix
   NN=malloc(h*sizeof(int*));
   for (i=0;i<h;i++)
       {
        N[i]=malloc(max*sizeof(int));
        NN[i]=malloc(max*sizeof(int));
       }

// diffusion matrix
   diff=malloc(2*sizeof(double*));
   for (i=0;i<2;i++) diff[i]=malloc(2*sizeof(double));
// 0 if the site i-th is occupied by herb, and >0 otherwise
   site=malloc(max*sizeof(int));
// 1 if the site is occupied by an ill tree, 0 otherwise
   ill=malloc(max*sizeof(int));
// tree in site i-th becomes symtomatic at a time tsym[i] after infection
   tsym=malloc(max*sizeof(double));
// tree in site i-th becomes hard at a time thar[i]
   thar=malloc(max*sizeof(double));


// 0 or 1 if the twig i-th in the site j-th is healthy or not
   ill_twig=malloc((Ntwig+1)*sizeof(int*));
   for (i=0;i<=Ntwig;i++) ill_twig[i]=malloc(max*sizeof(int));

// infection time of twig i-th in the site j-th
   tinf=malloc((Ntwig+1)*sizeof(double*));
   for (i=0;i<=Ntwig;i++) tinf[i]=malloc(max*sizeof(double));

// position of the k-th insect
   posix=malloc((Ninsect+1)*sizeof(int));

// twig of the branch in the site posix[j], where the j-th insect is on
   twig=malloc((Ninsect+1)*sizeof(int));

// health state of the j-th insect (0 healthy, 1 ill)
   port=malloc((Ninsect+1)*sizeof(int));



   for (i=0;i<max;i++)
       {
        site[i]=ill[i]=tsym[i]=thar[i]=0;
        for (j=1;j<=Ntwig;j++) tinf[j][i]=ill_twig[j][i]=0;
       }

   for (j=1;j<=Ninsect;j++)
       {
        posix[j]=port[j]=0;
        twig[j]=-10;
       }

// proportionality factor between the in-coming flux of infected insects and the
out-going one
   finf=malloc(h*sizeof(double));
// in-coming number of infected insects
   inf_out=malloc(h*sizeof(int));
// out-going number of infected insects
   inf_in=malloc(h*sizeof(int));

// time of the j-th insecticide treatments
```

```
   ti=malloc((Ninterv+1)*sizeof(int));
   ti[1]=t0;
   for (j=2;j<=Ninterv;j++) ti[j]=ti[j-1]+delta;
  }
static void init_next(void)
  {
   int i,j;

// LATTICE STRUCTURE ********************************************

// definition of nearest neighbours and second-nearest neighbours
   for (j=0;j<max;j++)
     {
       N[0][j]=j-1;
       N[1][j]=j-L;
       N[2][j]=j+1;
       N[3][j]=j+L;


       NN[0][j]=j-1-L;
       NN[1][j]=j+1+L;
       NN[2][j]=j-1+L;
       NN[3][j]=j+1-L;
     }
  }
static void init(void)
  {
   int i,j,ix,iy,s0,next;
   double x,p;

// PLACEMENT OF TREES ON THE LATTICE
******************************************************************

   Ne=max;
   Ntree=0;

// placement of olive tree center in the lattice site with abscissa and ordinate
ix%spaz=2 and iy%spaz=2
// each plant occupies all the nearest and the second-nearest neighbours of such
site

   for (i=0,s0=0;i<max;i++)
       {
// abscissa of the i-th site
            ix=i%L;
// ordinate of the i-th site
      iy=i/L;
        if ((ix%spaz==2)&&(iy%spaz==2))
           {
            s0++;
            site[i]=s0;
            Ne--;
            Ntree++;

// TREE ATTRIBUTES
********************************************************************************


// the time of symptom appearence, after infection, is randomly chosen from a
Gaussian distribution
            for (j=1;j<=10000000;j++)
                {
                 x=tsint+(double)(2*(int)(2.0*drand48())-1)*3.0*sigma*drand48();
                 p=exp(-(x-tsint)*(x-tsint)/(2.0*sigma*sigma));
```

```
                  if (drand48()<p)
                     {
                      tsym[i]=x;
                      break;
                     }
                 }
// the hardening time is randomly chosen from a Gaussian distribution

            for (j=1;j<=10000000;j++)
                 {
                  x=tind+(double)(2*(int)(2.0*drand48())-1)*3.0*sigmad*drand48();
                  if (x<0) x=0;
                  p=exp(-(x-tind)*(x-tind)/(2.0*sigmad*sigmad));
                  if (drand48()<p)
                     {
                      thar[i]=x;
                      break;
                     }
                 }
// tsym and thar are equal for each branch of the tree
            for(j=0;j<h;j++)
                {
                 next=N[j][i];
                 site[next]=s0;
                 Ne--;
                 tsym[next]=tsym[i];
                 thar[next]=thar[i];
                }
            for(j=0;j<h;j++)
                {
                 next=NN[j][i];
                 site[next]=s0;
                 Ne--;
                 tsym[next]=tsym[i];
                 thar[next]=thar[i];
                }

           }
        }
  }
void dynamics(void)
  {
   int i,j,k;
   int i0,j0,s0,s1,x,kh,next;
   int n1,n2,dead,u0,u1,ix,iy;
   double p,pcont;

   FILE *stream;
   char buffer[100];

// INTRODUCTION OF VECTORS ON THE LATTICE

// eclosion of Newborn insects at the current time nstep
//
   for (i=1;i<=Newborn;i++)
       {
        Nv++;
        x=(int)(max*drand48());
        posix[Nv]=x;
        if (site[x]>0)
          {
           j0=1+(int)(Ntwig*drand48());
           twig[Nv]=j0;
          }
```

```
        port[Nv]=0;

        if ((nstep<365)&&(Ninf0<Ninf))
           {
            Ninf0++;
            port[Nv]=1;
           }
       }

   for (i=0;i<h;i++) inf_in[i]=0;
// a number of insects equal to out arrive from outside
// out is the number of insects that went out in the previous time step
// the number of in-coming insects is equal to the number of out-going ones
   for (i=1;i<=out;i++)
        {
         Nv++;
         k=(int)(2.0*drand48());
         if (k==0)
            {
             ix=(L-1)*(int)(2.0*drand48());
             iy=(int)(L*drand48());
             if (ix==0) u0=0;
             else u0=2;
            }
         else
            {
             ix=(int)(L*drand48());
             iy=(L-1)*(int)(2.0*drand48());
             if (iy==0) u0=1;
             else u0=3;
            }
         x=ix+L*iy;
         posix[Nv]=x;
         if (site[x]>0)
            {
             j0=1+(int)(Ntwig*drand48());
             twig[Nv]=j0;
            }
// the number of infected in-coming insects on the edge u0 is equal to the
number of infected out-going ones on the same edge times finf[u0]
         if (inf_in[u0]<(finf[u0]*(double)inf_out[u0]))
          {
           port[Nv]=1;
           inf_in[u0]++;
          }
        }

   for (i=0,out=0;i<h;i++) inf_out[i]=0;


// VECTOR DYNAMICS AND DISEASE SPREADING

   if(Nv>0) for (j=0;j<48;j++)
       {
        for (i0=1,dead=0;i0<=Nv;i0++)
            {
// x is the position of insect i0
             x=posix[i0];
             s0=site[x];
             if (s0>0) //i0 is on the olive tree s0
                {
                  k=(int)(2.0*drand48());
//with probability 0.5 the vector i0 rests on the same branch and tries to move
to another twig
```

```
                  if (k==0)
                  {
// the vector i0 moves to another twig of the same branch
// posix does not change
// another twig is chosen

                  for (i=0;i<1000000;i++)
                     {
                      j0=1+(int)(Ntwig*drand48());
                      if (j0!=twig[i0]) break;
                     }

                  if (drand48()<ptt) twig[i0]=j0;
// the vector i0 moves from  twig[i0] to j0
                  else j0=twig[i0];
// the vector i0 does not move


// if the olive branch is tender, the vector eats
                  if ((nstep%365)<thar[x])
                      {
// if the twig is healthy and the vector is infected, the twig becomes infected
// with probability pm
                       if ((ill_twig[j0][x]==0)&&(port[i0]==1)&&(drand48()<pm))
                          {
                           ill[x]=ill_twig[j0][x]=1;

tinf[j0][x]=(double)nstep+(double)(i+1)/(double)(48*Nv);
                          }
// if the twig is infected and the vector is healthy, the vector becomes
infected with probability pcont=pp*(nstep-tinf)
                       if ((ill_twig[j0][x]==1)&&(port[i0]==0))
                          {
                           pcont=pp*(nstep-tinf[j0][x]);
                           if (pcont>1) pcont=1;
                           if (drand48()<pcont) port[i0]=1;
                          }

                       p=0;
// the vector dies after a bite with probability p
                       for (i=1;i<=Ninterv;i++) if ((nstep%365)>=ti[i])
p=pd*exp(-(double)((nstep%365)-ti[i])/tau);

                       if (drand48()<p)
                           {
                            posix[i0]=-10;
                            port[i0]=0;
                            dead++;
                           }
                       }
                    }
                 else
//the vector i0 moves to a nearest neighbour site
                    {
//a nearest neighbour site is randomly chosen
                     ix=x%L;
                     iy=x/L;

                     if (((nstep%365)>=thar[x])&&(ix%spaz!=2)&&(iy%spaz!=2))
                        {
//the olive branch is no more tender
//a direction, which allows the insect to leave the plant, is randomly chosen
                         for(i=0;i<1000000;i++)
                            {
```

```
                             kh=(int)(h*drand48());
                             next=N[kh][x];
                             s1=site[next];
                             if (s1==0) break;
                            }
                        }

//if the olive branch is tender
                     else
                        {
                         kh=(int)(h*drand48());
                         next=N[kh][x];
                         s1=site[next];
                        }

                     u0=1;//the departure site is on an olive tree
                     if(s1>0) u1=1; //the arrival site is on an olive tree
                     else u1=0; //the arrival site is on herb

                     p=diff[u0][u1];

                     if (s1>0) //the arrival site is on an olive tree
                         {
// a twig is randomly chosen
                          j0=1+(int)(Ntwig*drand48());
// if the olive tree in the arrival site is symtomatic the movement is rejected
                          if (nstep>=(tinf[j0][next]+tsym[next])) p=0;
                         }
// if the olive tree in the departure site is hard the movement is accepted with
probability 1
                      if ((nstep%365)>=thar[x]) p=1;

                      if (drand48()<p)
                         {

                          posix[i0]=next;
                          if (s1>0) twig[i0]=j0;
                         }
                      else
                         {
                          j0=twig[i0];
                          next=x;
                         }
                      if ((s1>0)&&((nstep%365)<thar[next]))
                         {
// the arrival site is on a tender olive
                          if ((ill_twig[j0][next]==0)
                             &&(port[i0]==1)&&(drand48()<pm))
                             {
                              ill[next]=ill_twig[j0][next]=1;

tinf[j0][next]=(double)nstep+(double)(i+1)/(double)(48*Nv);
                              }
                          if (ill_twig[j0][next]==1)
                             {
                              pcont=pp*(nstep-tinf[j0][next]);
                              if (pcont>1) pcont=1;
                              if((port[i0]==0)&&(drand48()<pcont)) port[i0]=1;
                              }
                          p=0;
                    for (i=1;i<=Ninterv;i++) if ((nstep%365)>=ti[i]) p=pd*exp(-
(double)((nstep%365)-ti[i])/tau);

                          if (drand48()<p)
```

```
                               {
                                posix[i0]=-10;
                                port[i0]=0;
                                dead++;
                               }
                            }
                       }
                    }
                if (s0==0)  //i0 is on herb
                    {
//a nearest neighbor site is randomly chosen

                     kh=(int)(h*drand48());
                     ix=x%L;
                     iy=x/L;

//since the lattice is open, if the departure site is on the lattice edge
//insects go out for some directions

                    if (((ix==0)&&(kh==0))||(ix==(L-
1)&&(kh==2))||((iy==0)&&(kh==1))||((iy==(L-1))&&(kh==3)))
                       {
                        posix[i0]=-10;
                        if (port[i0]>0) inf_out[kh]++;
                        port[i0]=0;
                        out++;
                        dead++;
                        continue;
                       }


                     next=N[kh][x];
                     s1=site[next];

                     u0=0; //the departure site is on herb
                     if(s1>0) u1=1; //the arrival site is on an olive tree
                     else u1=0; //the arrival site is on herb

                     p=diff[u0][u1];

                     if (s1>0) //the arrival site is on an olive tree
                         {
                          j0=1+(int)(Ntwig*drand48());
// if the olive branch in the arrival site is symtomatic the movement is
rejected
                          if (nstep>=(tinf[j0][next]+tsym[next])) p=0;
// if the olive branch in the arrival site is hard the movement is rejected
                          if ((nstep%365)>=thar[next]) p=0;
                         }

                    if (drand48()<p)
                       {
                        posix[i0]=next;

                        if (s1>0) //the arrival site is on an olive tree
                           {
                            twig[i0]=j0;
                            if ((ill_twig[j0][next]==0)
                                &&(port[i0]==1)&&(drand48()<pm))
                               {
                                ill[next]=ill_twig[j0][next]=1;

tinf[j0][next]=(double)nstep+(double)(i+1)/(double)(48*Nv);
                               }
```

```
                            if (ill_twig[j0][next]==1)
                               {
                                pcont=pp*(nstep-tinf[j0][next]);
                                if (pcont>1) pcont=1;
                                if((port[i0]==0)&&(drand48()<pcont)) port[i0]=1;
                               }
                            p=0;
                            for (i=1;i<=Ninterv;i++) if ((nstep%365)>=ti[i])
p=pd*exp(-(double)((nstep%365)-ti[i])/tau);

                            if (drand48()<p)
                               {
                                posix[i0]=-10;
                                port[i0]=0;
                                dead++;
                               }
                          }
                      }
                  }
              }
      if (dead>0)
           {
            for (n1=Nv;n1>0;n1--) if(posix[n1]<0) for (n2=n1+1;n2<=Nv;n2++)
                 {
                  posix[n2-1]=posix[n2];
                  port[n2-1]=port[n2];
                  twig[n2-1]=twig[n2];
                 }
            Nv=Nv-dead;
            for(n1=Nv+1;n1<=Nv+dead;n1++) port[n1]=0;
           }
       }

}

//**************************************** MAIN************************

int main(argc,argv)
int argc;
char **argv;
  {
   long int seed;
   int i,infected;
   double p,tsfarf;

   FILE *stream;
   char buffer[100];

   seed=984;
   seed+=(long)mype;

   srand48(seed);
// total number of lattice site
   max=L*L;
// number of nearest neighbours
   h=2*d;
// number of olive trees
   Ntree=(L-1)*(L-1)/(spaz*spaz);

   Nv=Ninf0=0;
   Ninsect=57600;

   memory_allocation();
```

```
   init_next();
   init();

// diffusion matrix

   diff[0][0]=1.0;
   diff[1][1]=pbb;
   diff[0][1]=1.0;
   diff[1][0]=phh;

// OPEN SYSTEM

   finf[0]=1.0;
   finf[1]=0.1;
   finf[2]=1.0;
   finf[3]=2.0;

   for (nstep=0;nstep<ST;nstep++)
      {
       for (i=0,infected=0;i<max;i++) if ((site[i]>0)&&(ill[i]>0)) infected++;

       sprintf(buffer, "inf_%i_%lg_%i_%i_%i",L,ptt,spaz,Ninterv,mype);
       if (nstep==0) stream = fopen(buffer, "w");
       else stream = fopen(buffer, "a");

       fprintf(stream,"%i %lg\n",nstep,(double)infected/(double)(9*Ntree));

       fclose(stream);


       if ((nstep%365)==0)
          {
           if (nstep!=0) Ninsect=(int)(0.09*(double)Nv);

           finf[1]=finf[1]*fact;
           finf[3]=finf[3]/fact;

           Nv=out=0;
           for (i=1;i<=Ninsect;i++) port[i]=0;

// the mean time to eclosion changes randomly from tecl-3 to tecl+3
           tsfarf=tecl+(2.0*(int)(2.0*drand48())-1)*(int)(4*drand48());
          }

       p=(1.0/sqrt(2.0*PIG*sigmas*sigmas))*exp(-((double)(nstep%365)-
tsfarf)*((double)(nstep%365)-tsfarf)/(2.0*sigmas*sigmas));

       Newborn=(int)((double)Ninsect*p);
// number of newborn per day at time nstep

       dynamics();
      }
    return 0;
  }
```
